# The use of generalized synthetic control method to evaluate air pollution control measures of G20 Hangzhou Summit

**DOI:** 10.3389/fpubh.2022.1021177

**Published:** 2022-10-03

**Authors:** Hao-Neng Huang, Zhou Yang, Yukun Wang, Chun-Quan Ou, Ying Guan

**Affiliations:** ^1^State Key Laboratory of Organ Failure Research, Department of Biostatistics, Guangdong Provincial Key Laboratory of Tropical Disease Research, School of Public Health, Southern Medical University, Guangzhou, China; ^2^Guangdong University of Foreign Studies South China Business College, Guangzhou, China

**Keywords:** air pollution, generalized synthetic control method, G20 Hangzhou Summit, causal inference, mega event

## Abstract

The traditional campaign-style enforcement in environmental governance has been debated whether its rebound effect is likely to eat away the short-term environmental benefits and subsequently bring about severer pollution. There are methodological challenges in assessing the effect of temporary environmental intervention. By applying the generalized synthetic control method (GSCM), we quantified and characterized the effectiveness of environmental regulations implemented for the G20 Hangzhou Summit held on 4–5 September, 2016. The summit was successful in reducing Air Quality Composite Index by 17.40% (95% CI: 9.53%, 24.60%), 13.30% (95% CI: 4.23%, 21.50%), and 10.09% (95% CI: 2.01%, 17.51%) in the core, strictly-regulated and regulated areas respectively, comparing with the index expected under a “No-G20” scenario during the preparatory period and the summit period (July–September 2016), and the reduction of the levels in specific pollutants (PM_10_, PM_2.5_, NO_2_, and CO) was also observed. Besides, the environmental benefits lasted for at least 3 months after the summit. This study demonstrates that the pollution control measures during the G20 Hangzhou Summit improved air quality immediately and continuously, and the GSCM provides a useful tool for evaluating the intervention effects of environmental regulations.

## Introduction

The problem of ambient air pollution has been highlighted because of its substantial health impacts and the ever-increasing public environmental awareness (1). The public is expecting stringent environmental governance. In addition to the long-term regulations, campaign-style enforcement of environmental interventions has gained popularity during mega event periods since the hosts are willing to burnish the country's international image and project its power in the region, and it achieved diverse results in different socioeconomic contexts. For example, the environmental enhancement measures for the Rio Olympics in Brazil resulted in reductions of most pollutants except O_3_, and the policy effects continued until 43 days after the Olympics (2). The primary goal of these measures was to yield immediate improvement in air quality and thus, obtain a blue sky during mega-events even at the cost of sacrificing some economic development or life convenience to an extent temporarily.

There are methodological challenges in assessing the effect of temporary environmental intervention. The interrupted time-series approach has been widely used to assess the intervention effect by comparing pollution levels between pre-intervention and post-intervention, while it is inapplicable when the intervention period is very short due to the lack of statistical power (3, 4). An alternative method commonly used is the traditional case-control design in which the critical issue is how to select a control unit matched with the treated unit. Since it is often difficult to find a single control unit that provides an appropriate comparison for the treated unit, we could use a weighted average of all control units in the donor pool to better approximate the pre-intervention characteristics of the treated unit, which is the basic concept of the synthetic control method (SCM) (5, 6). The generalized synthetic control method (GSCM) expands the SCM in several aspects. First, it adds to traditional SCM the possibility of calculating the treatment effect of multiple treated units simultaneously. Second, it improves the efficiency of the SCM and enhances its interpretability as it provides appropriate indicators of the uncertainty of estimates (e.g., confidence interval). Third, when the pre-treatment data is sufficient, a built-in cross-validation procedure automatically selects the proper number of factors and reduces the risk of over-fitting (7). To our best knowledge, although this method has been used in political science, economics, and clinical medicine (7–9), the GSCM has not been applied to the evaluation of environmental governance.

The 11th G20 Summit held on 4–5 September, 2016 in Hangzhou, China offered a golden opportunity to test the effectiveness of such interventions. This summit, involving leaders from 20 countries, was intended to bring the global economy onto the track of prosperity and stability and achieve sustainable and inclusive growth. To prepare for “the most important diplomatic event of the year at home,” Hangzhou and its neighboring cities in the Yangtze River Delta (YRD) region have made joint endeavors in restraining transboundary air pollution. Classified by the distances to the central court of the summit (50, 100, and 300 km), all cities within Zhejiang were divided into three types (i.e., the core areas, the strictly-regulated areas, and the regulated areas) which took different control measures. It is crucial to quantify the net effects of this intervention because these results will play an essential role in generalizing, revising, or canceling the interventions in the future.

The majority of these measures were short-term oriented, as they focused on restricting production or traffic and cessation of construction activities temporarily. However, it is worth noting that some efforts related to technological upgrades were also implemented due to this summit. Therefore, it was expected that the environmental benefits from hosting the G20 were not only limited to the duration of the summit but remained well beyond the summit.

Several studies have reported the undoubted short-term effect of this summit, including the reduction in the levels of criteria pollutants and gaseous precursors, inorganic and organic aerosols, and VOCs during the summit period *via* the WRF-Chem model, while the potential continuous impact of these control measures after the summit remained unknown (10–12). Besides, there was little literature on evaluating the effects in other cities in Zhejiang though they all responded to G20 environmental protection measures in varying degrees. Although composite indicators like the air quality index (AQI) can describe the overall effect, it is of great importance to figure out which pollutants are prone to be suppressed or rebounded due to the intervention. Previous studies showed inconsistent results when examining the effectiveness of emission control measures on O_3_. One study estimated a reduction of 11.7% in O_3_ level using a global chemical transport model during G20 in Hangzhou (13), and the drop was estimated to be 25.4% in another study using a Weather Research and Forecast and Community Multi-scale Air Quality model (11). However, in other two studies, O_3_ did not decrease or even increased (14, 15). The discrepancies in previous studies may result from the differences in the analytical methods used and the comparison period of interest.

In this study, using the GSCM in the evaluation of environmental regulations, we aim to quantify the effects of policy intervention related to the G20 Summit on the overall air quality and the levels of specific pollutants in different periods.

## Materials and methods

### Data

**Figure 1:**
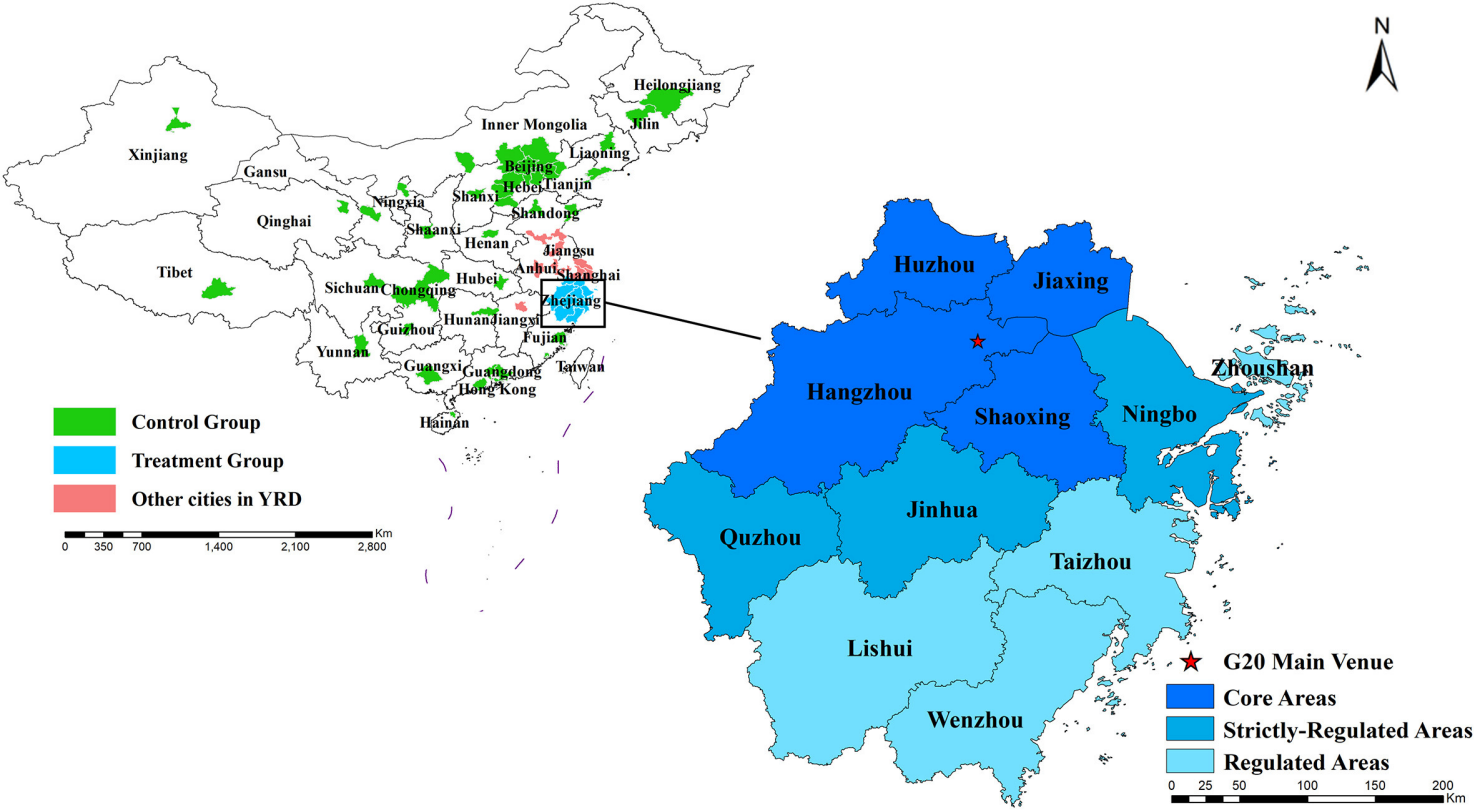
The distribution of 60 cities involved in this study.

In this study, the Air Quality Composite Index (AQCI) was used as the primary outcome. Therefore, we considered all 74 cities with AQCI data available before 2018 provided by the Ministry of Ecology and Environment of China (https://www.mee.gov.cn/hjzl/dqhj/cskqzlzkyb/). Among 74 cities (11 cities in Zhejiang as treated cities and 63 cities as potential control cities), 14 control cities were excluded where the meteorological data were unavailable from the China Meteorological Data Center. Finally, our study was based on 49 control cities (Supplementary Table 1) and 11 treated cities including four cities (Hangzhou, Jiaxing, Huzhou, and Shaoxing) in the core areas, three (Ningbo, Jinhua, and Quzhou) in the strictly-regulated areas and four (Lishui, Taizhou, Wenzhou, and Zhoushan) in the regulated areas (Figure 1). The monthly data of six primary air pollutants were collected from the China Air Quality Online Monitoring and Analysis Platform (https://www.aqistudy.cn/) from July 2014 to December 2016. The AQCI is a non-dimensional index that has been widely used to reflect urban air quality. The China Ministry of Ecology and Environment ranks the air quality of the primary cities every month according to AQCI (16, 17). Compared to the air quality index (AQI) which only reflects the level of the single primary pollutant with the highest individual air quality index, AQCI integrates the information of six primary pollutants (SO_2_, NO_2_, PM_10_, PM_2.5_, CO, and O_3_) (18). Moreover, AQI cannot convey definite information about the seriously-polluted situation since the Chinese air pollution system does not report the actual level of AQI beyond 500. Thus, we considered monthly AQCI instead of AQI as one of the primary outcomes. In brief, the calculation of AQCI is as follows: First, calculate the single index of each air pollutant i from Ci/Si, where Ci represents monthly average concentrations for SO_2_, NO_2_, PM_10_, PM_2.5_, or the 95th percentile of daily mean level for CO or the 90th percentile of daily maximum 8-h level for O_3_; Si means corresponding of annual average concentrations of the national ambient air quality secondary standards in China (GB3095-2012, Supplementary Table 2). Then, the AQCI was calculated by adding up six indexes.

Meteorological impacts on air pollution have been well-documented (19). In this study, 4 monthly meteorological factors (average rainfall, average wind speed, average temperature, and average relative humidity) were selected which were calculated as the average of daily data collected from the China Meteorological Data Center (http://data.cma.cn/). Considering the potential effect of other environmental and socioeconomic factors on air quality reported in literature and the availability of data (20, 21), we obtained relevant data from the China City Statistical Yearbook (2014–2016), including GDP per capita, green area, and population size which account for the economic development level and the productivity of a city, environmental purification capacity and the emitting behavior in terms of consumption. Considering the heteroscedasticity of data, we transformed the measurements into their natural logarithm form except for rain and temperature because of zero and negative values (20). Although the raw data suggested a decline in AQCI in June 2016, to avoid data-driven specification of the starting time of intervention, we pre-specified July 2016 as the starting point according to official documentation, since the 2016 Air Pollution Prevention and Control Implementation Plan issued by the Department of Ecological and Environment of Zhejiang Province listed many measures for the air pollution control related to the G20 Hangzhou Summit with the majority of the rectification and reform deadlines being set at the end of June 2016 (22). The inclusion of June 2016 in the control period may lead to a conservative estimation of the intervention effect. The pre-treatment periods were defined as July 2014–June 2016. And we defined two different treatment period: one was restricted to July 2016-September 2016 (preparatory period and summit period) to investigate the short-term effect of treatment, the other treatment period was extended to the end of 2016 (i.e., July 2016-December 2016, including preparatory period, summit period, and post-summit period), to examine the persistence of the intervention effect.

### Method

In this study, we used the GSCM to estimate the policy effect of the G20 Hangzhou Summit on air quality. It constructs the counterfactual based on a linear interactive fixed effects model (LIFEM) that allows for the heterogeneity of treatment effects across units and time. The model can be expressed as:


$$Y_{it}=\delta_{it}D_{it}+x_{it}{}^{{}^{\prime }}\beta +\lambda_{i}^{{}^{\prime }}f_{t}+\varepsilon_{it}$$

where *Y*_*it*_ is the logged average level of the single pollutant or AQCI in each city i in month t, *D*_*it*_ is the treatment indicator with 0 and 1 indicating pre-treatment (before July 2016) and post-treatment (in or after July 2016) for the treated units and with 0 at all time (July 2014–December 2016) for the control units, and δ_*it*_ means the heterogeneous treatment effect; *x*_*it*_ is a vector of observed covariates including meteorological (i.e., rainfall, wind speed, temperature, and relative humidity) and environmental and socioeconomic factors (i.e., GDP per capita, green area, and population size), and β is the corresponding coefficients; *f*_*t*_ represents unobserved common factors and λ_i_ is unknown factor loadings; ε_*it*_ is external shocks for city i in month t and follows the normal distribution with a mean of zero. Based on the potential outcome framework, our main purpose is to calculate the average treatment effect on the treated (ATT):


$$\begin{array}{l} ATT_{t}=\text{exp}\left(\frac{1}{N_{tr}}\underset{i\in T}{\sum }\left[Y_{it}\left(1\right)-{Ŷ}_{it}\left(0\right)\right]\right)-1 \end{array}$$

where *N*_*tr*_ is the number of treated cities. Since *Y*_*it*_(1) can be observed in the post-treatment period, we could estimate the counterfactual *Y*_*it*_(0) as: ${Ŷ}_{it}\left(0\right)=X_{it}^{{}^{\prime }}\hat{\beta }+{\hat{\lambda }}_{i}^{{}^{\prime }}{\hat{f}}_{t}$, in which $\hat{\beta \;}$and ${\hat{f}}_{t}$ is obtained from a LIFEM only using all control cities data with some constraints on *f*_*t*_, and λ_*i*_. The ${\hat{\lambda }}_{i}^{{}^{\prime }}$ is calculated from the other LIFEM by minimizing the mean squared error (MSE) of the pre-treatment outcome for the treated cities. ATT represents the percent change of AQCI or specific pollutant concentrations in different areas compared to the counterfactual in the “no-G20” scenario. The 95% confidence intervals and the *P*-value were estimated using a parametric bootstrap procedure. A built-in cross-validation scheme was used to automatically select the optimal number of unobserved factors of the LIFEM that produces the lowest MSE, which minimizes the risk of overfitting and is easy to implement (7). Root mean squared error (RMSE), mean absolute error (MAE), and mean absolute percent error (MAPE) were used to examine the appropriateness and accuracy of the counterfactual.

Since the 2010 Shanghai World Expo, if any mega-events are held in YRD, the authorities of surrounding cities will issue some short-term environmental regulations for fear of emissions transport to ensure the host city's air quality. Therefore, the cities under the YRD regional joint prevention and control strategy including Shanghai and cities in Jiangsu, Anhui, and Jiangxi province were removed from the donor pool in the main analysis. In the sensitivity analysis, these cities were included to explore the net effect of control measures in the treated cities under study and clarify the robustness of the main results. Additionally, we performed the other sensitivity analysis by adjusting the treatment timing backward to examine whether the estimated causal effects are consistent (i.e., whether the estimated factors and loadings dramatically change when we shorten the pre-treatment periods a little).

We performed the analysis using the package of *gsynth* in R 4.1.1. A two-sided *P* < 0.05 was considered as statistical significance.

## Results

**Figure 2:**
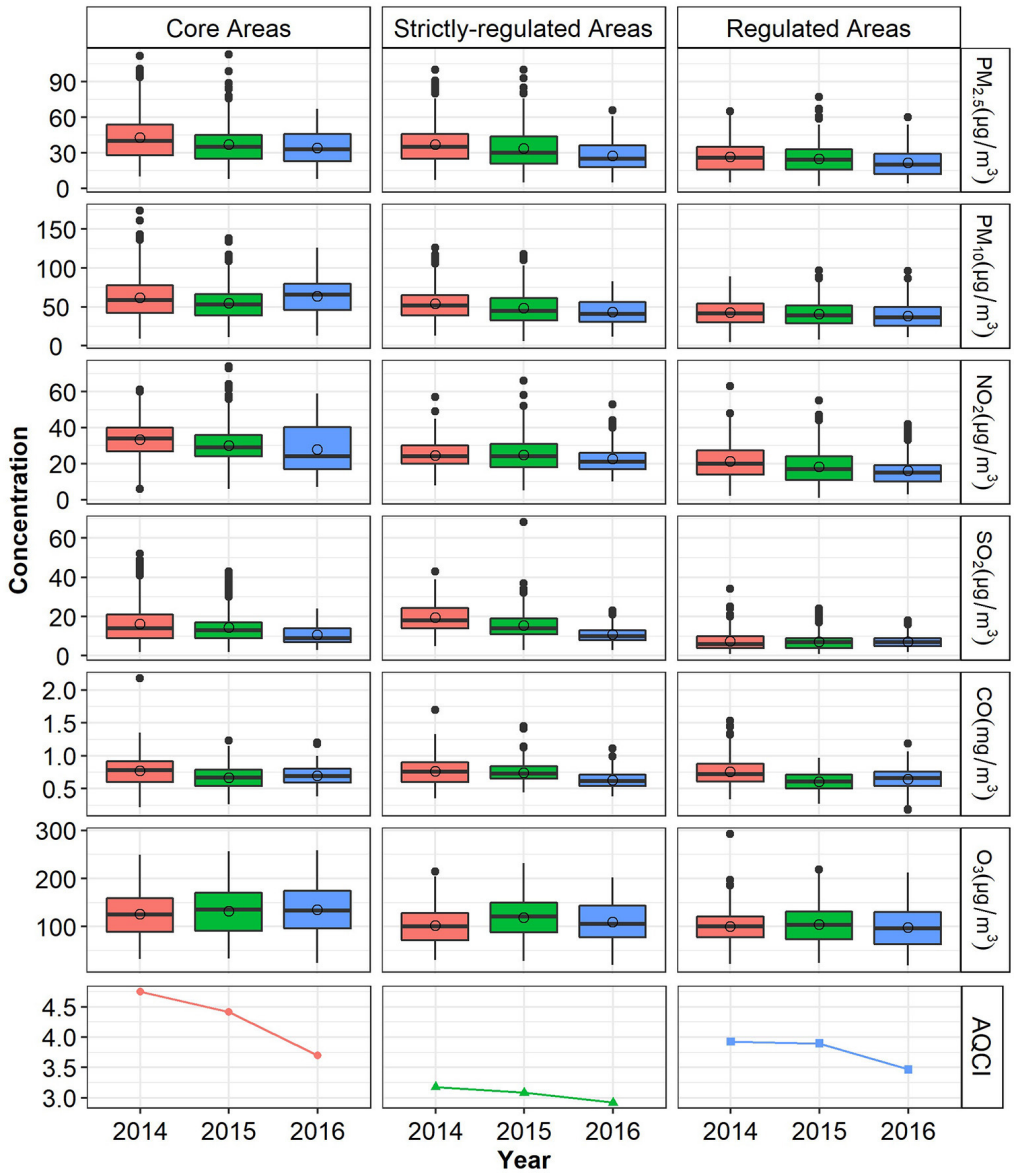
AQCI and main air pollutants concentration before and during the G20 period. Time span: July–September. The open circle represents the mean.

Figure 2 shows the levels of the AQCI and six pollutants in three areas (core, highly-regulated and regulated areas). Compared with the same period in 2014 and 2015, July to September 2016 witnessed a downward trend in AQCI and the average monthly concentrations for most air pollutants in all areas, especially for PM_2.5_, NO_2_, and SO_2_, except that the concentrations of PM_10_ and O_3_ in the core areas were higher than those in 2014 and 2015. During the G20 period (September 4–5, 2016) in the core area, the observed daily mean concentrations of PM_2.5_, PM_10_, NO_2_, SO_2_, and CO were 36.75, 65.75, 19.63, 8.75μg/m^3^, 0.65mg/m^3^ respectively, all well below national air quality standards (Supplementary Table 2), whereas the level of O_3_ (140.25 μg/m^3^) almost exceeded the standard (160 μg/m^3^).

**Figure 3:**
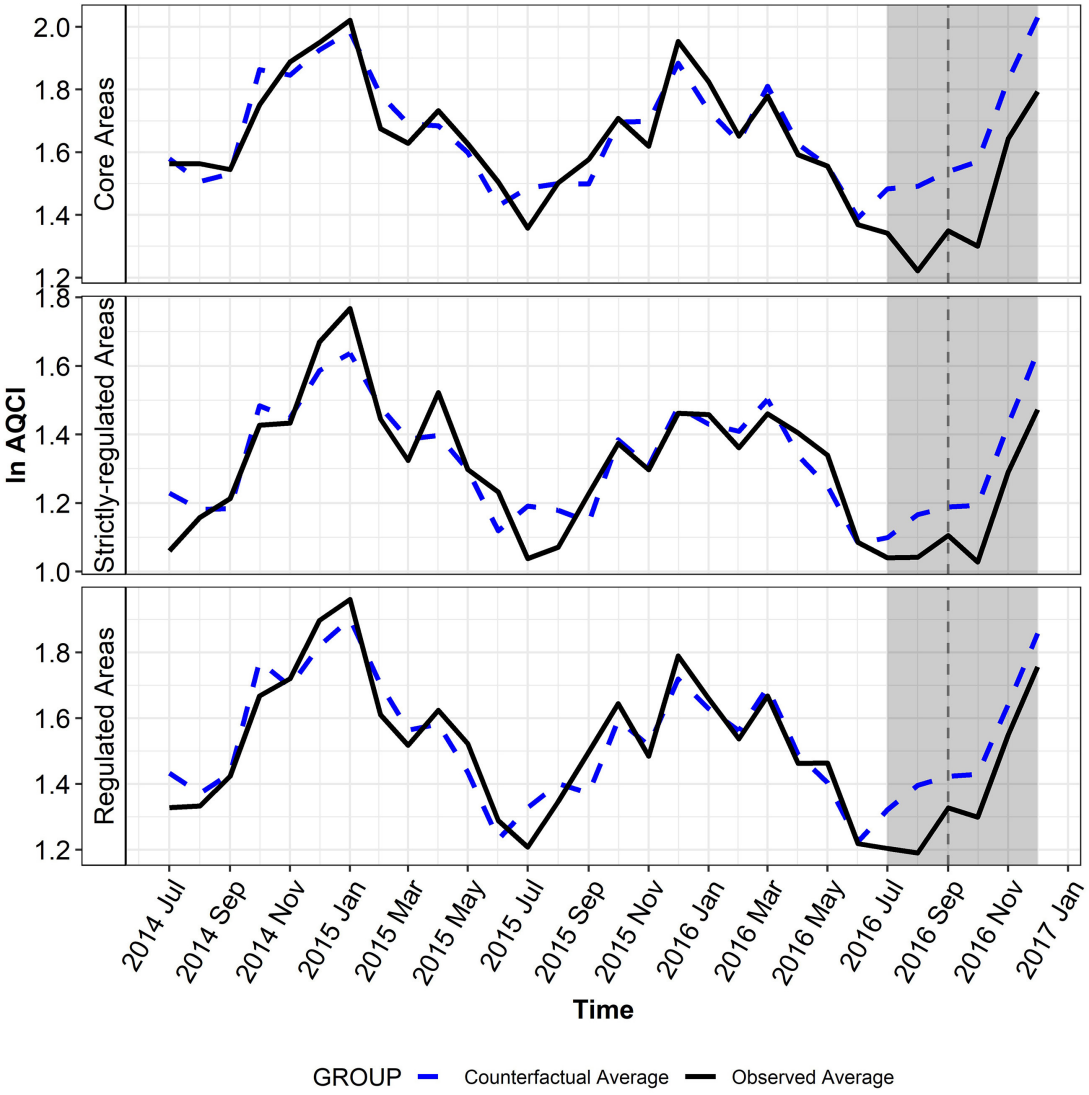
The observed and counterfactual monthly average of ln AQCI. The treatment period is shaded in gray and the vertical dashed line presents the time of the summit.

Figure 3 illustrates that the GSCM estimator fitted AQCI well in the pre-intervention period (July 2014–June 2016). The MAPE ranged from 4.4 to 6.4% and the RMSE was 0.079–0.113 in three types of areas (Supplementary Table 3). From July to September 2016, there was an average reduction of 17.40% (95% CI: 9.53%, 24.60%) in AQCI in the core areas when comparing the actual level to the counterfactual that represents the business-as-usual scenario, and AQCI also declined by 13.30% (95% CI: 4.23%, 21.50%) and 10.09% (95% CI: 2.01%, 17.51%) in the strictly-regulated areas and the regulated areas, respectively. Significant benefits from the summit-related environmental regulations were also observed for three pollutants (PM_2.5_, PM_10_, and NO_2_), with a decrease of 18.23% (95% CI: 6.30%, 28.64%), 21.58% (95% CI: 8.18%, 33.03%), and 24.51% (95% CI: 13.06%, 34.47%), respectively (Table 1). The reduction of PM_2.5_ and CO was also noticeable in the strictly-regulated areas, with a decrease of 20.12% (95% CI: 6.25%, 31.93%) and 13.17% (95% CI: 0.93%, 23.89%), respectively. Similar results were obtained from the regulated areas, although the reduction rate was relatively lower.

**Table 1:** The effect (ATT with 95% CI) of policy on AQCI and six main pollutants in three types of area.

**Treatment period**	**Pollutant**	**Core areas**	**Strictly-regulated areas**	**Regulated areas**
Jul–Sep 2016	AQCI	−17.40* (−24.60, −9.53)	−13.30* (−21.50, −4.23)	−10.09* (−17.51, −2.01)
	PM_2.5_	−18.23* (−28.64, −6.30)	−20.12* (−31.93, −6.25)	−15.46* (−26.21, −3.14)
	PM_10_	−21.58* (−33.03, −8.18)	−10.34 (−24.47, 6.41)	−12.23 (−24.31, 1.78)
	NO_2_	−24.51* (−34.47, −13.06)	−12.58 (−27.73, 5.74)	−23.97* (−34.03, −12.37)
	SO_2_	−17.68 (−37.93, 9.20)	−17.16 (−36.49, 8.05)	−17.49 (−36.00, 6.36)
	CO	−10.77 (−21.33, 1.21)	−13.17* (−23.89, −0.93)	−10.76 (−21.30, 1.19)
	O_3_	−9.94 (−21.15,2.87)	−9.89 (−23.94, 6.77)	−4.35 (−17.16, 10.43)
Jul–Dec 2016	AQCI	−19.43* (−29.33, −8.13)	−11.68 (−23.99, 2.63)	−14.27* (−24.73, −2.36)
	PM_2.5_	−22.68* (−35.22, −7.71)	−15.87 (−30.62, 2.02)	−19.14* (−32.59, −2.98)
	PM_10_	−20.26* (−33.26, −4.72)	−4.27 (−24.79, 21.86)	−16.46* (−29.65, −0.80)
	NO_2_	−18.94* (−31.23, −4.45)	−10.53 (−27.06, 9.75)	−16.44* (−29.71, −0.67)
	SO_2_	−21.27* (−36.19, −2.85)	−17.65 (−35.22, 4.69)	−14.08 (−29.33, 4.47)
	CO	−8.97 (−19.68, 3.17)	−12.55 (−25.23, 2.28)	−6.43 (−17.82, 6.55)
	O_3_	−9.50 (−22.14, 5.19)	−8.75 (−24.93, 10.90)	2.36 (−14.79, 22.96)

^*^Significant at 5% level.

AQCI, air quality composite index; ATT, average treatment effect on the treated.

Table 1 presents the effect of policy on AQCI and six main pollutants till December (i.e., 3 months after the summit). Although the time span of post-treatment was extended, a significant reduction of 19.43% (95% CI: 8.13%, 29.33%) was still found in the AQCI in the core areas. The PM_2.5_, PM_10_ concentration in these areas decreased by 22.68% (95% CI: 7.71%, 35.22%) and 20.26% (95% CI: 4.72%, 33.26%), respectively. For NO_2_ and SO_2_, the reduction level was also noticeable. In addition, the regulated areas witnessed significant decreases in AQCI, PM_2.5_, PM_10_, and NO_2_ while all decreases in the strictly-regulated areas were not statistically significant.

In the sensitivity analyses, when reconstructing the control group with the inclusion of other cities in YRD, the G20 Hangzhou Summit still had a positive impact on the air quality in three types of areas. Specifically, the estimated reductions in AQCI were 16.10% (95% CI: 8.35%, 23.21%), 10.42% (95% CI: 1.29%, 18.71%), and 8.89% (95% CI: 0.58%, 16.51%) during July to September in the core, strictly-regulated and regulated areas, respectively (Supplementary Table 4). In addition, backdating the intervention for 1 or 2 months (i.e., May or June) does not obviously change the estimated ATT after July 2016, and the ATT before July 2016 maintained stably at a very low and non-significant level (Supplementary Table 5), reassuring the appropriateness of treatment timing we pre-defined.

## Discussion

To our knowledge, this is the first study using the GSCM to evaluate the effectiveness of the environmental intervention. The model specification of the GSCM is a crucial issue. We considered various covariates, including meteorological measures and GDP, population size and green area in each city in the model. The weighted combination of 49 control cities fitted the treated cities well during the pre-treatment period, indicating that the selection of the control group is appropriate and the construction of the counterfactual is reasonable. Our study verifies that the GSCM offers a valid tool for environmental program evaluation, especially when the commonly used interrupted time-series approach or the traditional case-control design is inapplicable. For example, in this study, the post-intervention time points were only 3 or 6, which was far from adequate in terms of statistical power (23–25).

Our study indicated the overall air quality in Hangzhou was improved during the summit period, which was consistent with previous studies although the effect size differed since they were based on different study periods. For instance, a simulation-based study integrated with the WRF-CMAQ model showed the predicated concentrations of PM_2.5_ were reduced by 56% from September 4 to September 5, 2016 in Hangzhou (11). Another study suggested the SO_2_, NO_2_, PM_10_, and PM_2.5_ concentrations in Hangzhou decreased by 42.6, 57.1, 36, and 38.5% respectively compared to the same period (August 24–September 6) from the 5 preceding years (15). Moreover, different from previous studies, our study was based on a larger spatial and temporal scale and attempted to evaluate the policy effect comprehensively. We further revealed that the positive impacts could be traced back to July and last until December, and all cities in Zhejiang in addition to Hangzhou gained the G20 benefits in varying degrees since. In general, more substantial and persistent improvement in AQCI was observed in the core areas than in the strictly-regulated and regulated areas.

The remarkable benefits were primarily attributable to stringent short-term environmental governance. Since August 24, 2016, a large-scale shutdown of factories had begun in Hangzhou and surrounding areas. It's noteworthy that citizens in Hangzhou were arranged to enjoy a 7-day holiday since 1 September, and they were encouraged to travel outside Hangzhou. The summer vacation of 2016 was also postponed to September 8, later than the summit. Since the benefits from these stringent short-term measures cannot be sustained, more attention should be paid to the long-term-oriented measures implemented for the summit. To control three categories of pollution (industrial pollution, coal-fired pollutants, and vehicle exhaust), specific actions have been taken successively during the preparatory period of the summit since July 2016, including retrofitting projects related to volatile organic compounds (VOCs) in polluting industries (i.e., coating, printing), expanding the restricted areas that disallow the combustion of high-polluting fuels, technological upgrades in five key sectors (i.e., steel, cement, chemical engineering, petrochemical, and non-ferrous metal), renewal of high-emission vehicles and replacing fossil fuels with clean energy and so on. As a result, continuous and remarkable improvements in AQCI from July to December 2016 were consistently observed in the core and the controlled areas. It suggests that, stimulated by mega-events, governments can achieve long-lasting environmental benefits only by incorporating some short-term governance with some long-term environmental regulation policies from the perspective of sustainability.

Our study highlighted the importance of joint prevention and control of atmospheric pollutants considering the transboundary nature of air pollution. To ensure blue skies in Hangzhou during the summit, other YRD cities surrounding Zhejiang had also released plans to restrict air pollution before G20. For instance, about 255 manufacturers in Shanghai related to petrochemical, steel, and cement were projected to temporarily reduce their production or shut down. We observed air quality improvement in three treatment areas including 10 cities in Zhejiang other than Hangzhou, and our sensitivity analyses revealed that the estimated AQCI reductions in three areas became smaller once nine YRD cities outside Zhejiang are included in the control group, indicating that YRD joint prevention and control measures played a certain role in fighting for G20 Blue Sky Protection Campaign. Regardless of targeting local or regional improvement of air quality, this cooperation needs to be strengthened in the future since long-range transport may offset the local emission control effects to some extent (26).

A few empirical studies have provided controversial evidence on the effectiveness of the G20 emission control measures during the summit period (13, 15). In our study, based on the causal framework of GSCM, we did not detect a significant reduction of O_3_, which may be due to the O_3_-VOCs-NO_x_ sensitivity pattern after implementing emission control measures. A previous study has shown that O_3_ in urban Hangzhou mainly presents VOC-limited in summer (27). That is to say, if NO_x_ emission control measures are stronger than the VOCs emission control measures in these areas, the inhibitory effect of NO_x_ on O_3_ production will be weak, and finally, the O_3_ concentration will rise up. Additionally, our results did show a substantial reduction in the NO_2_ concentration. Similarly, during the 2014 APEC period in Beijing, the regional reduction in VOCs was significantly less than NO_x_, which increased the O_3_ concentration during the APEC conference (28–30). The sources of O_3_ precursors are complex, with a wide variety of species. The conventional PM_2.5_-oriented air quality regulations may deteriorate O_3_ levels because PM_2.5_ could serve as a scavenger of NO_x_ and hydroperoxy radicals which may otherwise react with NO to produce O_3_ (31, 32). Therefore, a joint control strategy for PM_2.5_ and O_3_ is necessary and requires further investigation.

The results reported herein should be considered in light of some limitations. We investigated the net effects of the G20 Summited-related joint pollution control measures on air quality in 11 cities in Zhejiang. However, the spillover effects for YRD cities were not assessed, leading to underestimating the total effect of the intervention. In addition, isolating the effect of the G20 Summit on pollutant concentrations is challenging since pollution levels are also influenced by meteorological conditions and other factors. Our study attempted to address the impacts of weather and some socioeconomic factors on pollution levels by incorporating them in the model. However, we cannot avoid the possibility that some other influencing factors were not observed. Besides, since a series of intervention measures have been taken and evaluated together, the contribution of each specific measure to the environmental benefits needs further investigation.

## Conclusion

The air pollution control measures implemented for the G20 Summit exerted remarkable improvement in AQCI and reduction in the levels of specific pollutants (PM_2.5_, PM_10_, NO_2_, and CO). Thankfully, the G20 Blue Sky is not a temporary show, and the constant improvement of air quality was observed to last at least 3 months after the summit, which is mainly attributed to a series of stringent policies restricting emissions and technical upgrades. This causal effect was estimated using the GSCM, which provides a useful tool for evaluating the intervention effects of environmental regulations.

## Data availability statement

Publicly available datasets were analyzed in this study. This data can be found at: The AQCI data are available from the Ministry of Ecology and Environment (https://www.mee.gov.cn/hjzl/dqhj/cskqzlzkyb/) and air quality data are accessible from the China Air Quality Online Monitoring and Analysis Platform (https://www.aqistudy.cn/). Meteorological data are available from the China Meteorological Data Center (http://data.cma.cn/) upon reasonable request.

## Author contributions

C-QO: conceptualization, supervision, and funding acquisition. YG: conceptualization and supervision. H-NH: data curation, writing—original draft preparation, and visualization. ZY and YW: writing—reviewing and editing. All authors read and approved the final manuscript.

## Funding

This work was supported by the National Nature Science Foundation of China (81973140).

## Conflict of interest

The authors declare that the research was conducted in the absence of any commercial or financial relationships that could be construed as a potential conflict of interest.

## Publisher's note

All claims expressed in this article are solely those of the authors and do not necessarily represent those of their affiliated organizations, or those of the publisher, the editors and the reviewers. Any product that may be evaluated in this article, or claim that may be made by its manufacturer, is not guaranteed or endorsed by the publisher.

## Abbreviations

AQI: air quality index
AQCI: air quality composite index
ATT: average treatment effect
GSCM: generalized synthetic control method
LIFEM: linear interactive fixed effects model
MSE: mean squared error
SCM: synthetic control method
YRD: Yangtze River Delta.
