# Supplementary material for: The use of generalized synthetic control method to evaluate air pollution control measures of G20 Hangzhou Summit
Source: Front Public Health. 2022 Oct 3;10:1021177. doi: 10.3389/fpubh.2022.1021177 (PMC9574187; doi:10.3389/fpubh.2022.1021177)
Supplement: Supplementary file 1 [file Table_1.DOCX]

Supplementary Material

**Table of contents**

[**Table A1** The list of control group. 2](#_Toc111574030)

[**Table A2** Chinese ambient air quality standards (GB3095-2012). 3](#_Toc111574031)

[**Table A3** Summary of model fitting in pre-treatment period. 4](#_Toc111574032)

[**Table A4** Short-term effect (ATT with 95% CI) of policy on AQCI and six main pollutants, estimated by additionally effects including nine YRD cities in the donor pool. 5](#_Toc111574033)

[**Table A5** The effect (ATT with 95% CI) of policy on AQCI, estimated by changing the start month of treatment. 6](#_Toc111574034)

# Table A1 The list of control group.

| **Control Group (49 cities)** | | | |
| --- | --- | --- | --- |
| **Municipality** | Beijing  Shanghai*  Tianjin  Chongqing | **Jiangsu** | Xuzhou*  Lianyungang*  Huaian*  Nanjing*  Nantong*  Changzhou*  Suzhou* |
| **Guangdong** | Guangzhou  Shenzhen  Huizhou  Jiangmen | **Hebei** | Shijiazhuang  Zhangjiakou  Chengde  Qinhuangdao  Langfang  Tangshan  Baoding  Xingtai |
| **Fujian** | Xiamen  Fuzhou | **Shandong** | Qingdao  Jinan |
| **Heilongjiang** | Haerbin | **Anhui** | Hefei* |
| **Liaoning** | Dalian  Shenyang | **Sichuan** | Chengdu |
| **Jilin** | Changchun | **Henan** | Zhengzhou |
| **Shanxi** | Taiyuan | **Hubei** | Wuhan |
| **Shaanxi** | Xianyang | **Hunan** | Changsha |
| **Qinghai** | Xining | **Jiangxi** | Nanchang* |
| **Gansu** | Lanzhou | **Guangxi** | Nanning |
| **Xizang** | Lasa | **Guizhou** | Guiyang |
| **Xinjiang** | Wulumuqi | **Yunnan** | Kunming |
| **Ningxia** | Yinchuan | **Hainan** | Haikou |
| **Inner Mongolia** | Huhehaote |  |  |

*Indicates cities in YRD which were not included in the main analysis.

# Table A2 Chinese ambient air quality standards (GB3095-2012).

| **Pollutant** | **Concentration Limit** | | **Unit** | | **Average Time** |
| --- | --- | --- | --- | --- | --- |
|  | Ⅰ* | Ⅱ* |  |  |  |
| PM_2.5_ | 35 | 75 | | μg/m^3^ | 24-hour average |
| PM_10_ | 50 | 150 | | μg/m^3^ | 24-hour average |
| NO_2_ | 80 | 80 | | μg/m^3^ | 24-hour average |
| SO_2_ | 50 | 150 | | μg/m^3^ | 24-hour average |
| O_3_ | 100 | 160 | | μg/m^3^ | Maximum 8-hour average |
| CO | 4 | 4 | | mg/m^3^ | 24-hour average |

*The functional zoning of ambient air is divided into two categories. The category Ⅰ includes areas that require special protection such as nature reserves and scenic spots; the category Ⅱ includes residential areas, commercial areas, industrial areas and rural areas.

**Table A3** Summary of model fitting in pre-treatment period.

| **Study Period*** | **Areas** | **MAE** | **MSE** | **RMSE** | **MAPE** |
| --- | --- | --- | --- | --- | --- |
| Jul-Sep  2016 | Core Areas | 0.066 | 0.008 | 0.088 | 0.044 |
|  | Strictly-Regulated Areas | 0.066 | 0.006 | 0.079 | 0.046 |
|  | Regulated Areas | 0.067 | 0.007 | 0.083 | 0.054 |
| Jul-Dec  2016 | Core Areas | 0.083 | 0.013 | 0.113 | 0.055 |
|  | Strictly-Regulated Areas | 0.070 | 0.007 | 0.083 | 0.049 |
|  | Regulated Areas | 0.079 | 0.010 | 0.100 | 0.064 |

* We built the model using two different study periods in which the pre-treatment periods were both July 2014-June 2016.

**Table A4** Short-term effect (ATT with 95% CI) of policy on AQCI and six main pollutants, estimated by additionally effects including nine YRD cities in the donor pool.

| **Pollutant** | **Core Areas** | | | **Strictly-regulated Areas** | | **Regulated Areas** | |  |
| --- | --- | --- | --- | --- | --- | --- | --- | --- |
|  | **40 controls** | **49 controls** | **40 controls** | | **49 controls** | **40 controls** | **49 controls** | |
| **AQCI** | -17.40*  (-24.60, -9.53) | -16.10*  (-23.21, -8.35) | -13.30*  (-21.50, -4.23) | | -10.42*  (-18.71, -1.29) | -10.09*  (-17.51, -2.01) | -8.89*  (-16.51, -0.58) | |
| **PM_2.5_** | -18.23*  (-28.64, -6.30) | -19.72*  (-30.25, -7.60) | -20.12*  (-31.93, -6.25) | | -9.70  (-21.50, 3.88) | -15.46*  (-26.21, -3.14) | -12.62*  (-23.52, -0.17) | |
| **PM_10_** | -21.58*  (-33.03, -8.18) | -9.90  (-20.48, 2.08) | -10.34  (-24.47, 6.41) | | -7.49  (-19.73, 6.63) | -12.23  (-24.31, 1.78) | -8.94  (-19.81, 3.41) | |
| **NO_2_** | -24.51*  (-34.47, -13.06) | -13.79  (-28.49, 3.93) | -12.58  (-27.73, 5.74) | | -2.97  (-23.60, 23.24) | -23.97*  (-34.03, -12.37) | -7.62  (-25.27, 14.21) | |
| **SO_2_** | -17.68  (-37.93, 9.20) | -3.77  (-27.35, 27.47) | -17.16  (-36.49, 8.05) | | -10.13  (-32.67, 19.95) | -17.49  (-36.00, 6.36) | -10.38  (-30.04, 14.81) | |
| **CO** | -10.77  (-21.33, 1.21) | -10.54  (-21.96, 2.54) | -13.17*  (-23.89, -0.93) | | -12.93  (-26.12, 2.60) | -10.76  (-21.30, 1.19) | -4.67  (-16.66, 9.05) | |
| **O_3_** | -9.94  (-21.15,2.87) | -1.51  (-14.38, 13.30) | -9.89  (-23.94, 6.77) | | -11.94  (-24.67, 2.95) | -4.35  (-17.16, 10.43) | -3.09  (-15.31, 10.90) | |

*Significant at 5% level; AQCI: air quality composite index; ATT: average treatment effect on the treated

**Table A5** The effect (ATT with 95% CI) of policy on AQCI, estimated by changing the start month of treatment.

| Area | Time | Start month of treatment | | | | |
| --- | --- | --- | --- | --- | --- | --- |
|  |  | 2016-07 | | 2016-06 | | 2016-05 |
| Core Areas | 2016-05 | -0.13 (-10.56,11.51) | -0.36 (-11.01, 11.56) | | -0.40 (-12.63, 13.54) | |
|  | 2016-06 | -2.13 (-11.61,8.35) | -2.43 (-13.22, 9.71) | | -2.47 (-13.89, 10.47) | |
|  | 2016-07 | -13.20 (-24.44,-0.29) | -13.48 (-24.96, -0.24) | | -13.52 (-25.06, -0.21) | |
|  | 2016-08 | -23.67 (-34.36,-11.24) | -23.77 (-34.31, -11.54) | | -23.80 (-34.7, -11.09) | |
|  | 2016-09 | -17.21 (-27.92,-4.9) | -17.32 (-28.06, -4.97) | | -17.35 (-28.34, -4.67) | |
|  | 2016-10 | -23.62 (-35.54,-9.5) | -23.80 (-35.87, -9.46) | | -23.83 (-36.22, -9.02) | |
|  | 2016-11 | -17.24 (-34.35,4.33) | -17.50 (-34.86, 4.47) | | -17.53 (-35.05, 4.73) | |
|  | 2016-12 | -21.07 (-38.76,1.73) | -21.26 (-39.3, 2.14) | | -21.27 (-39.24, 2.02) | |
|  | Average* | -19.43 (-29.33,-8.13) | -17.35 (-26.83, -6.65) | | -15.43 (-24.93, -4.74) | |
| Strictly-regulated Areas | 2016-05 | 6.10(-6.46, 20.36) | 6.03 (-6.85, 20.70) | | 6.77 (-8.01, 23.92) | |
|  | 2016-06 | -0.60(-11.26, 11.35) | -0.68 (-12.85, 13.19) | | 0.03 (-12.6, 14.48) | |
|  | 2016-07 | -11.17(-24.37, 4.32) | -11.25 (-24.54, 4.38) | | -10.57 (-23.99, 5.23) | |
|  | 2016-08 | -18.65(-31.35, -3.59) | -18.67 (-31.42, -3.57) | | -18.08 (-31.2, -2.45) | |
|  | 2016-09 | -9.13(-22.42, 6.44) | -9.16 (-22.54, 6.53) | | -8.63 (-22.4, 7.58) | |
|  | 2016-10 | -12.24(-27.79, 6.66) | -12.29 (-28.22, 7.16) | | -11.85 (-28.22, 8.24) | |
|  | 2016-11 | -8.84(-30.1, 18.87) | -8.92 (-30.86, 19.97) | | -8.53 (-30.81, 20.94) | |
|  | 2016-12 | -9.64(-32.72, 21.36) | -9.70 (-33.34, 22.32) | | -9.46 (-32.92, 22.19) | |
|  | Average* | -11.68(-23.99, 2.63) | -10.24 (-22.03, 3.35) | | -7.81 (-19.59, 5.7) | |
| Regulated Areas | 2016-05 | 8.86 (-0.43, 19.01) | 8.83 (-0.93, 19.55) | | 11.77 (-0.93, 26.09) | |
|  | 2016-06 | -0.16 (-8.84, 9.35) | -0.19 (-10.68, 11.53) | | 1.80 (-9.55, 14.57) | |
|  | 2016-07 | -6.46 (-18.93, 7.93) | -6.49 (-19.05, 8.03) | | -4.17 (-16.77, 10.34) | |
|  | 2016-08 | -12.83 (-24.67, 0.88) | -12.84 (-24.94, 1.22) | | -10.67 (-22.86, 3.45) | |
|  | 2016-09 | -12.36 (-24.33, 1.49) | -12.38 (-24.59, 1.82) | | -10.24 (-22.9, 4.51) | |
|  | 2016-10 | -16.44 (-28.88, -1.83) | -16.47 (-29.05, -1.67) | | -13.09 (-26.82, 3.22) | |
|  | 2016-11 | -16.1 (-32.79, 4.73) | -16.16 (-33.22, 5.26) | | -11.15 (-29.68, 12.27) | |
|  | 2016-12 | -20.78 (-40.1, 4.78) | -20.82 (-40.34, 5.09) | | -16.36 (-36.73, 10.57) | |
|  | Average* | -14.27 (-24.73, -2.36) | -12.42 (-22.38, -1.18) | | -6.89 (-17.01, 4.46) | |

Average*: the average treatment effect on the treated cities within the whole treatment period; AQCI: air quality composite index; ATT: average treatment effect on the treated
